# Supplementary material for: Clinical Utility of Ghrelin-O-Acyltransferase (GOAT) Enzyme as a Diagnostic Tool and Potential Therapeutic Target in Prostate Cancer
Source: J Clin Med. 2019 Nov 22;8(12):2056. doi: 10.3390/jcm8122056 (PMC6947219; doi:10.3390/jcm8122056)
Supplement: Supplementary file 1 [file jcm-08-02056-s001.pdf]

Supplementary Figures:

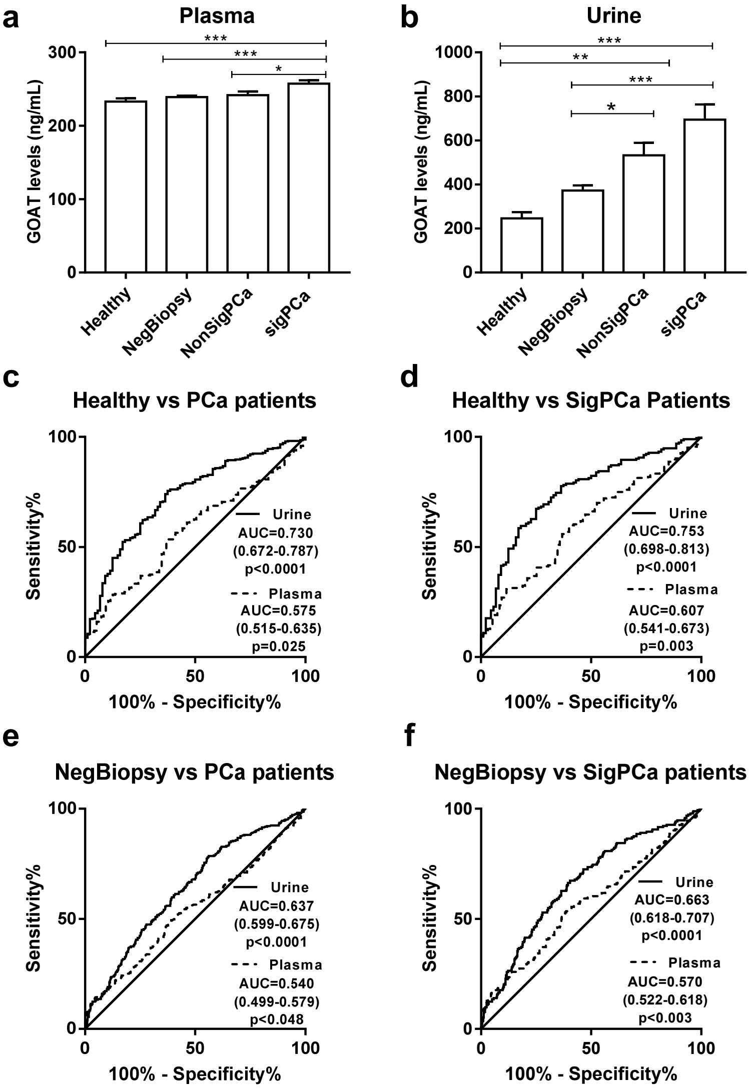

Supplemental Figure S1. GOAT levels in urine and plasma from patients with and without PCa. Levels of GOAT in plasma (**a**) and urine (**b**) of healthy individuals (*n* = 97), patients with suspect of PCa but negative result in the biopsy (NegBiopsy; *n* = 549), patients diagnosed with non-significant PCa (NonSigPCa; *n* = 143) and patients diagnosed with significant PCa (SigPCa; *n* = 204). Data represent mean ± SEM. c-f) Comparison of the receiver operating characteristic (ROC) curves analyses of urine (solid line) and plasma GOAT (dashed line) capacity to discriminate between healthy individual and PCa patients (**c**), healthy individuals and SigPCa patients (**d**), NegBiopsy and PCa patients (**e**), or NegBiopsy and SigPCa patients (**f**). Asterisks (*, $p < 0.05$; **, $p < 0.01$, ***, $p < 0.001$) indicate values that significantly differ between groups.

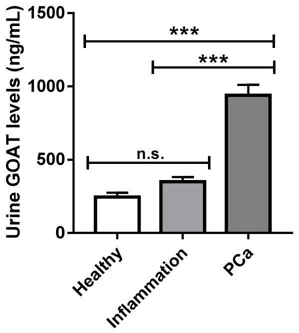

Supplemental Figure S2. Comparison of urine GOAT levels among healthy individuals (n = 97), patients with prostatic inflammatory diseases (n = 211) and PCa patients (n = 347). Data represent mean ± SEM. Asterisks (***, $p < 0.001$) indicate values that significantly differ between groups. n.s.: non statistically significant differences.

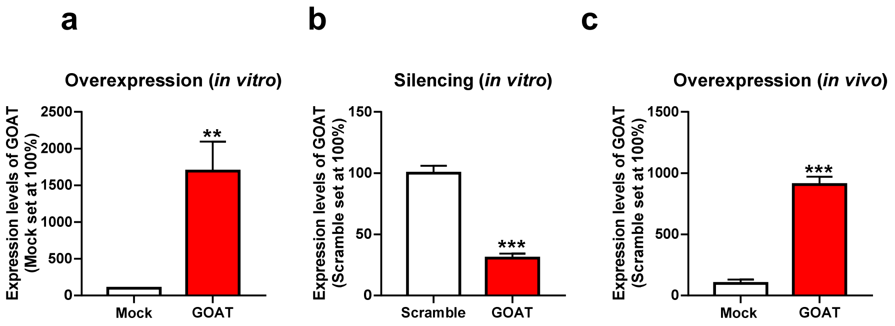

Supplemental Figure S3. Validation of *GOAT* overexpression (**a**) and silencing (**b**) in DU145 cells. (**c**) Validation of *GOAT* overexpression in the xenograft tumors. mRNA levels were determined by qPCR and adjusted by a normalization factor (calculated with the expression levels of *ACTB* and *GAPDH* using GeNorm). Data represent percentage of control cells (mock or scramble; mean ± SEM). Asterisks (**, $p < 0.01$, ***, $p < 0.001$) indicate values that significantly differ between groups.

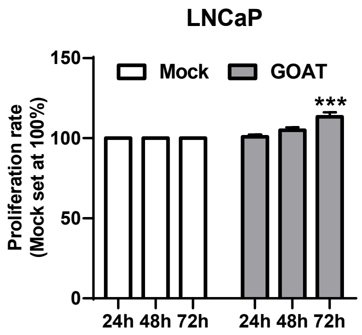

Supplemental Figure S4. Cell proliferation rate (determined by Alamar-Blue assay) at 24, 48 and 72 h in response to *GOAT* overexpression in LNCaP cells. One experiment was carried out (n = 1) with four technical replicates. Results are referred as percentage of mock. Statistically differences were calculated using the technical replicates. Asterisks (***, $p < 0.001$) indicate values that significantly differ between groups.
